# Cardiotoxicity Associated With Chimeric Antigen Receptor (CAR)-T Cell Therapy for Hematologic Malignancies: A Systematic Review

**DOI:** 10.7759/cureus.28162

**Published:** 2022-08-19

**Authors:** Kerollos S Hanna, Harkirat Kaur, Mohammad S Alazzeh, Abhay Thandavaram, Aneeta Channar, Ansh Purohit, Bijay Shrestha, Deepkumar Patel, Hriday Shah, Lubna Mohammed

**Affiliations:** 1 General Physician, California Institute of Behavioral Neurosciences & Psychology, Fairfield, USA; 2 Orthopaedic Surgery, California Institute of Behavioral Neurosciences & Psychology, Fairfield, USA; 3 Internal Medicine, California Institute of Behavioral Neurosciences & Psychology, Fairfield, USA; 4 Research, California Institute of Behavioral Neurosciences & Psychology, Fairfield, USA; 5 Family Medicine, California Institute of Behavioral Neurosciences & Psychology, Fairfield, USA; 6 Neurology, California Institute of Behavioral Neurosciences & Psychology, Fairfield, USA

**Keywords:** car-t cell therapy heart adverse events, cardiac complication with immunotherapy for hematologic malignancies, heart complications with immunotherapy, chimeric antigen receptor cardiotoxicity, car-t cell therapy cardiotoxicity

## Abstract

Chimeric Antigen Receptor (CAR)-T cell therapy has been one of the most important breakthroughs for treating hematologic malignancies. On the other hand, the therapy had many toxicities. One of the toxicities of the CAR-T therapy is cardiotoxicity. The goal of the systematic review is to elaborate on the cardiotoxicities related to CAR-T therapy for hematologic malignancies. The systematic review is following the Preferred Reporting Items for Systematic Review and Meta-Analyses (PRISMA) 2020 guidelines. The systematic search was done using PubMed, PubMed Central (PMC), Google Scholar, Cochrane Library, ScienceDirect, and clinicaltrial.gov. The search and selection of studies were done on April 28, 2022, and May 6, 2022, respectively. The studies were selected based upon participants, intervention, and outcomes (PIO) elements and the articles that were included were, full-text articles published within the last ten years, clinical trials, meta-analyses, randomized controlled trial, review, and systematic review. The exclusion criteria were non-hematologic malignancy, non-English-language articles. The initial search had 2,159 publications. The publications were assessed with assessment tools of Scale of the Assessment of Narrative Review Articles (SANRA), Newcastle-Ottawa Scale (NCOS), and Cochrane Collaboration Risk of Bias Tool (CCRBT), which led to selection of eight publications. The systematic review concludes that cardiotoxicity happened in adults and pediatric patients receiving the CAR-T cell therapy and that those cardiac adverse events had many risk factors. Therefore, monitoring these cardiotoxicities is highly essential.

## Introduction and background

Cancer treatment has taken eminent progress over the past 30 years. The treatment has grown into targeted immunotherapy from other modalities such as surgery, radiation, and chemotherapy [1]. One of the immunotherapies is chimeric antigen receptor (CAR)-T cell therapy. CAR-T cells are autologous genetically modified T cells capable of expressing CAR, which is a protein that is modified to detect tumor antigens leading to activation of T cells and destruction of targeted tumor cells [1,2]. The steps of manufacturing CAR-T cells are leukapheresis, which is removing T cells from the blood, genetically modifying the T cells to express specific CAR, culturing the modified T cells to reach a specific dose, performing lymphodepletion to the recipient of CAR-T cell therapy, and then reinfusing the cells into the patient. The genetic modification of the isolated T cells happens by transduction with retroviral or lentiviral vector containing the CAR [2,3].

After 2012, when Whitehead was treated with CAR-T cell therapy from relapsing acute lymphocytic leukemia, the therapy has been approved to treat pediatric patients with acute lymphocytic leukemia (ALL). Also, the number of clinical trials for CAR-T therapy has gone over 180 clinical trials all over the world [4]. Moreover, CAR-T cell therapy has shown efficacy in treating hematologic malignancies such as acute lymphocytic leukemia, non-Hodgkin lymphoma, and multiple myeloma [2].

Even though the CAR-T cell therapy has an eminent response in refractory hematologic malignancies and a response rate around 50-93% with relapsed/refractory (r/r) mantle cell lymphoma, there were multiple dangerous adverse events for the therapy such as neurotoxicity and cytokines release syndrome (CRS), which is reported in retrospective studies. CRS is a systemic inflammatory response that results from the release of interleukin (IL)-6 leading to hypoxia, hypotension, and end-organ dysfunction. As there are several studies that shed the light on these toxicities; there are fewer data on cardiotoxicities that accompany the CAR-T cell therapy, which are defined as all cardiovascular side effects that happen with the cancer therapy [1,5-8]. Cardiovascular problems are more likely to occur in cancer patients as they are more likely to have preexisting cardiovascular problems or cardiovascular complications due to previous cancer therapy. The complications have been reported in 10-36 % of patients receiving CAR-T cell therapy [1,7]. In addition, cardiotoxicities associated with CAR-T cell therapy has been mentioned in retrospective studies for adult and children population. For example, in Burstein at el.'s study, 24 of 98 pediatric patients (24%) had hypotension and were medicated with inotropic for the adverse event and underwent echocardiogram, which showed left systolic ventricular dysfunction in 10 of these patients (50%). Heart complications could differ in severity and reversibility from pediatric to adult patients [7]. The goal of the systematic review is to summarize the associated cardiotoxicities with CAR-T therapy used for hematologic malignancies.

## Review

Methods

The systematic review is done based on the Preferred Reporting Items for Systematic Review and Meta-Analyses (PRISMA) 2020 guidelines [9].

Eligibility Criteria 

The selection of the studies according to the participants, intervention, and outcomes (PIO) elements: participants include patients with hematologic malignancies; intervention includes CAR-T therapy recipients; and outcome includes cardiotoxicity. Additional inclusion and exclusion criteria were added as well. Full-text articles published within the last ten years, clinical trials, meta-analyses, randomized controlled trial, review, and systematic review were in the criteria for inclusion, and non-hematologic malignancy and non-English-language articles were in the criteria for exclusion.

The data search was done systematically using PubMed, PubMed Central (PMC), Google Scholar, Cochrane, clinicaltrial.gov, and ScienceDirect. The search was done on April 28, 2022. The duplicate papers were excluded using Microsoft Excel 2021. The search strategy and keywords are illustrated in Table 1.

**Table 1:** Illustrate Keywords and Search Strategy. CAR: Chimeric antigen receptor; PMC: PubMed Central

Databases	Keywords	Search strategy	Filters	Search results
PubMed	CAR-T cell therapy cardiotoxicity	Hematologic Malignancy OR blood malignancy OR Lymphoma OR Leukemia OR Multiple Myeloma OR "Hematologic Neoplasms/therapy"[Mesh]) OR "Leukemia/therapy"[Mesh]) OR "Lymphoma/therapy"[Mesh]) OR "Multiple Myeloma"[Mesh] AND CAR-T Therapy OR Chimeric antigen receptor OR "Immunotherapy, Adoptive/adverse effects"[Mesh] OR "Immunotherapy, Adoptive/mortality"[Mesh] OR "Immunotherapy, Adoptive/pharmacology"[Mesh] OR "Immunotherapy, Adoptive/therapeutic use"[Mesh] OR "Immunotherapy, Adoptive/therapy"[Mesh] ) AND Cardiotoxicity, Cytokine release syndrome OR "Cardiotoxicity/analysis"[Mesh] OR "Cardiotoxicity/epidemiology"[Mesh] OR "Cardiotoxicity/etiology"[Mesh] OR “Cardiotoxicity/mortality"[Mesh] OR "Cardiotoxicity/pathology"[Mesh] OR "Cardiotoxicity/physiology"[Mesh] OR "Cardiotoxicity/physiopathology"[Mesh] OR "Cardiotoxicity/prevention and control"[Mesh]	Last 10 years, full text	1,974
PMC	CAR-T cell therapy cardiotoxicity, Chimeric antigen receptor Cardiotoxicity	Hematologic Malignancy OR blood malignancy OR Lymphoma OR Leukemia OR Multiple Myeloma OR "Hematologic Neoplasms/therapy"[Mesh]) OR "Leukemia/therapy"[Mesh]) OR "Lymphoma/therapy"[Mesh]) OR "Multiple Myeloma"[Mesh] AND CAR-T Therapy OR Chimeric antigen receptor OR "Immunotherapy, Adoptive/adverse effects"[Mesh] OR "Immunotherapy, Adoptive/mortality"[Mesh] OR "Immunotherapy, Adoptive/pharmacology"[Mesh] OR "Immunotherapy, Adoptive/therapeutic use"[Mesh] OR "Immunotherapy, Adoptive/therapy"[Mesh] ) AND Cardiotoxicity, Cytokine release syndrome OR "Cardiotoxicity/analysis"[Mesh] OR "Cardiotoxicity/epidemiology"[Mesh] OR "Cardiotoxicity/etiology"[Mesh] OR “Cardiotoxicity/mortality"[Mesh] OR "Cardiotoxicity/pathology"[Mesh] OR "Cardiotoxicity/physiology"[Mesh] OR "Cardiotoxicity/physiopathology"[Mesh] OR "Cardiotoxicity/prevention and control"[Mesh]	Last 10 years, full text	56
ScienceDirect	CAR-T cell therapy cardiotoxicity, Chimeric antigen receptor Cardiotoxicity	CAR-T cell therapy cardiotoxicity	2012-2022 research articles, encyclopedia, review articles.	27
Google Scholar	CAR-T cell therapy cardiotoxicity, heart complications with immunotherapy	CAR-T cell therapy cardiotoxicity OR heart complication with CAR-T cell Therapy	2012-2022	100
Cochrane Library	CAR-T cell therapy cardiotoxicity, cardiac complication with immunotherapy for hematologic malignancies	CAR-T cell therapy cardiotoxicity OR heart complication with CAR-T cell Therapy OR cardiac complications with immunotherapy	May 2012- May 2022	0
clinicaltrials.gov	CAR-T cell therapy cardiotoxicity, CAR-T cell therapy heart adverse events	CAR-T cell therapy cardiotoxicity OR chimeric antigen receptor T-Cell therapy heart complications	All age groups: children, adults, and older adults, male and female. Studies with or without results. Interventional studies, observational studies, patient registries, expanded access.	2

Results

**Figure 1:**
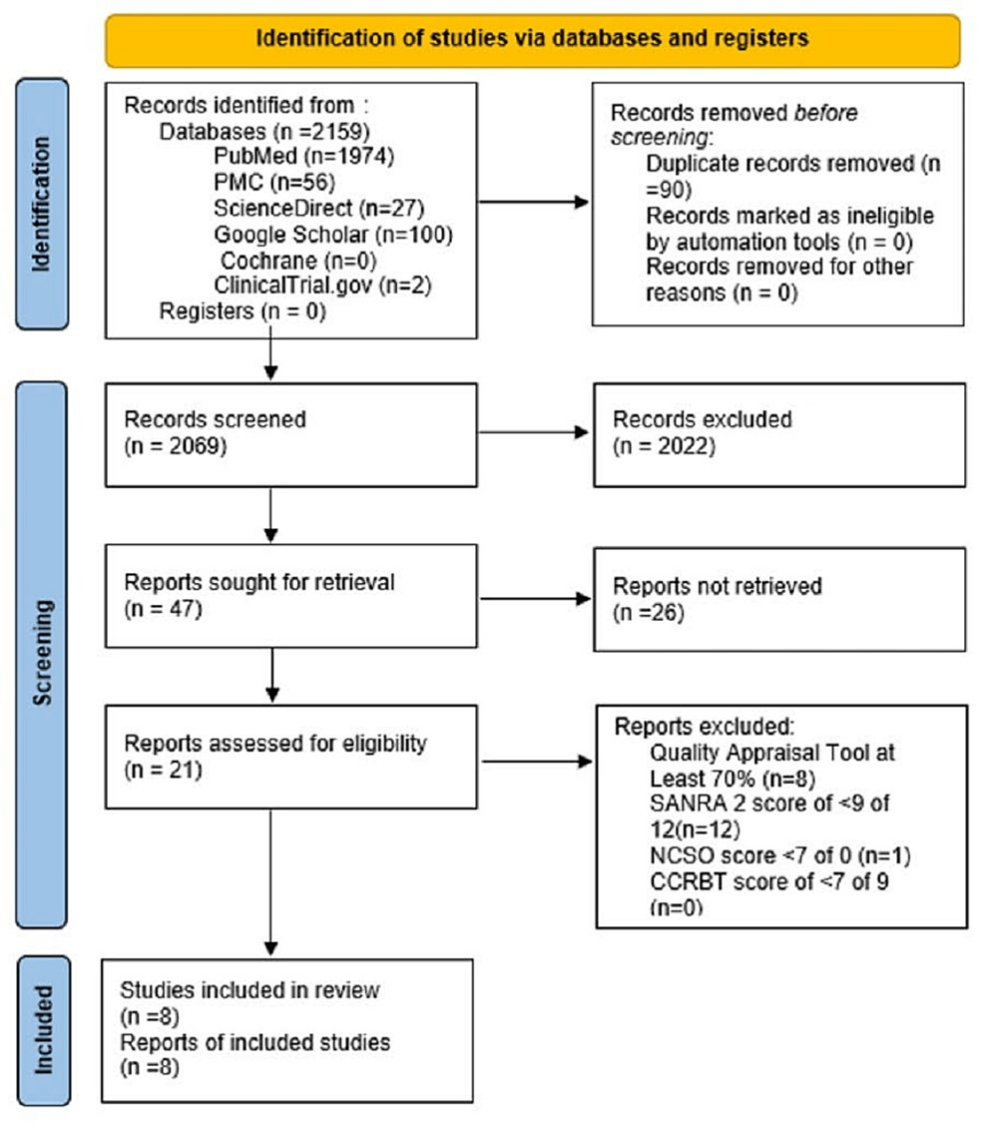
Flow chart showing selection process of studies NCOS: Newcastle-Ottawa Scale; SANRA: Scale of the Assessment of Narrative Review Articles; CCRBT: Cochrane Collaboration Risk of Bias Tool, PMC: PubMed Central

Data search was done, and 2,159 publications had relevant titles. Duplicate removals were done, and 2,069 articles remained. Publications’ titles and abstracts were screened based on PIO, eligibility criteria, and inclusion and exclusion criteria, and 21 publications remained. Quality appraisal for publications was done based upon Scale of the Assessment of Narrative Review Articles (SANRA), Newcastle-Ottawa Scale (NCOS), and Cochrane Collaboration Risk of Bias Tool (CCRBT), which resulted in the exclusion of 13 publications, and eight publications remained. A flow diagram shows the screening process in Figure 1. Also, the characteristics of accepted studies is mentioned in Table 2. 

**Table 2:** Illustration of characteristics of studies accepted in the review CD: Cluster of differentiation; CAR: Chimeric antigen receptor; JACC: Journal of the American College of Cardiology

First author, Year	Study name	Study type	Study population	Therapy	Outcome
Patel et al., 2021 [1]	Cardiovascular toxicities of CAR T-cell therapy	Review	Not applicable	CAR-T cell therapy	CAR-T cell therapy results in cardiac adverse events, especially when the treated patients usually has preexisting cardiac problems.
Totzeck et al., 2022 [2]	Cardiotoxicity from chimeric antigen receptor-T cell therapy for advanced malignancies	Review	Not application	Chimeric antigen T cell therapy	Heart-adverse events associated with the CAR-T cell therapy
Baik et al., 2021 [3]	Mechanisms of cardiovascular toxicities associated With immunotherapies	Review	Not applicable	Immunotherapies	Immunotherapy results in cardiotoxicities that more research needs to elaborate on in the future.
Gantra et al., 2019 [4]	Chimeric antigen receptor T-Cell therapy for cancer and heart: JACC council perspectives	Review	Not applicable	CAR-T cell therapy	CAR-T cell therapy is an epic novel therapy for malignancies and better understanding of toxicities and cardiotoxicities is important to increase therapies effectiveness.
Burns et al., 2021 [5]	Cardiotoxicity associated with anti-CD19 chimeric antigen receptor T-Cell (CAR-T) therapy: recognition, risk factors, and management	Review	Not applicable	Anti-CD19 CAR-T cell therapy	CD19 CAR-T cell therapy treats refractory hematologic malignancies. Cardiotoxicities happens in pediatric and adult population treated with the therapy. More studies need to address the associated Cardiotoxicities due to the limited data available.
Alvi et al., 2019 [6]	Cardiovascular events among adults treated with chimeric antigen receptor T-cells (CAR-T)	Retrospective observational study	137 patients	CAR-T cell therapy	In the adult population, cardiovascular adverse events are common after CAR-T cell therapy.
Lefebvre et al., 2020 [7]	Cardiovascular effects of CAR-T cell therapy: a retrospective study	Retrospective observational study	145 patients	CAR-T therapy	Subjects who are treated with CAR-T cell therapy are prone to cardiotoxicity and should undergo cardiovascular monitoring.
Briasoulis et al., 2022 [8]	Cardiotoxicity of non-anthracycline cancer chemotherapy agents	Review	Not applicable	Non-anthracycline chemotherapy	Cardiotoxicities are associated with chemotherapy and immunotherapy, resulting in early stopping of the therapy. The goal of the medical team to ensure no early termination or stop of the therapy due to heart toxicities.

Discussion

Cardiovascular Toxicities of CAR T-Cell Therapy

There are several Food and Drug Administration (FDA)-approved CAR-T cell therapies with significant response rates. However, there are many cardiotoxicities that happen in pediatric and adult populations, which will be covered in the following sections.

CAR-T Cell Therapies Approved by the FDA

The FDA approved two CAR-T cell therapies, which were tisagenlecleucel and axicabtagene ciloleucel, in 2017. Tisagenlecleucel is a CAR-T cell therapy that targets cluster of differentiation (CD)-19, which is approved to treat relapsed and refractory B-cell acute lymphocytic leukemia in pediatrics and young adults. Later, the therapy had another approval to treat diffuse large B-cell lymphoma. Axicabtagene ciloleucel therapy is a CD-19 targeting therapy that is approved to treat refractory mantle cell lymphoma. Brexucabtagene autoleucel was approved by the FDA to treat refractory mantle cell lymphoma in July 2020 and lisocabtagene maraleucel was approved to treat refractory large cell lymphoma in February 2021 [1,10,11].

Response of Patients to CAR-T Cell Therapy

The response rate for the approved CAR-T cell therapy was significant. Axicabtagene ciloleucel, which was given to 101 adult patients diagnosed with refractory large B cell lymphoma in ZUMA-1 phase clinical trial, had an 84% overall response. Additionally, the therapy had a 45% survival rate at six months and 52% at 18 months. Regarding brexucabtagene autoleucel, which was given to 256 adult patients with refractory large B cell lymphoma, it had a 93% of overall response in ZUMA -2 phase II clinical trial. Moreover, the therapy had a 67 % complete response rate at six months and a 61% progression-free survival rate at 12 months. Also, lisocabtagene maraleucel was given to 256 patients diagnosed with refractory large B-cell lymphoma and showed a 73% complete response rate in TRANSCEND phase I clinical trial. The treated individuals showed a 75% overall survival rate at six months and a 58% survival rate at 12 months [1].

CAR-T Cell Therapy Complications

Even though the CAR-T therapy showed an amazing response rate, it had many adverse events such as neurotoxicity, cytokine release syndrome, and cardiac adverse events [1,8]. There are various cardiac adverse events associated with CAR-T infusion such as heart arrest, high troponin level and ventricular dysfunction which will be elaborated upon in the following sections [12].

Pathophysiology of Cardiotoxicity

Cardiotoxicity is any heart complication that happens to patients with CAR-T therapy [8]. Heart complications that happen with CAR-T cell therapy are related to CRS which has multiple grades based on the Common Terminology Criteria for Adverse Events (CTCAE) scale. The grades of CRS according to the CTCAE scale are illustrated in Table 3. CRS severity is related to CAR-T cell therapy dose and tumor burden [3,5,13]. The CRS and associated heart complications happen when the infused CAR-T cells recognize the tumor cells. The CAR-T cells release several cytokines: IL-1 and IL-2, interferon-gamma, tumor necrosis factor-alpha, and IL-6, which activate prostaglandins. The activation of prostaglandins causes many symptoms such as tachycardia, hypotension, and multiorgan failure. Moreover, the cytokines released specifically IL-6 have a role in recruiting more T cells and the CRS, and the higher the level of IL-6, the more significant the cardiac adverse event [5,8,14,15].

**Table 3:** Illustration of the CRS grading according to CTCAE scale Sources:  [3,5,11,13] CRS: Cytokine release syndrome; CTCAE: Common Terminology Criteria for Adverse Events

CRS	CTCAE
Grade I: mild	Mild reaction and tachycardia
Grade II: moderate	Moderate responsive hypotension to fluid infusion, arrhythmia
Grade III: severe	Arrhythmia nonstable, hypotension, shock that needs several vasopressor medications
Grade IV: life-threatening	Resistant shock to treatment, left ventricular ejection fraction less than 20%, need for a ventilator, life-threatening arrhythmia
Grade V: death	Death

Risk Factors of Cardiotoxicity

There are multiple risk factors for cardiotoxicities in adults and pediatric populations, such as CRS occurrence, troponin level, age, previous heart dysfunction, high lipids, and others. Table 4 shows the risk factors for the pediatric and adult populations [5]. One of the important risk factors as illustrated in Table 4 is elevated troponin level. Alvi et al.'s retrospective study found that there was a relationship between elevation of troponin and cardiovascular adverse events [1,4,5,6, 16].

**Table 4:** Risk factors for cardiotoxicities in adults and young age Source: [5] CAR: Chimeric antigen receptor; CRS: Cytokine release syndrome

Pediatric and young adults	Adults
Blast more than 25 percent in bone marrow prior to treatment	CRS Grade III or more
Prior diastolic heart dysfunction	Old age
Low ejection fraction prior to CAR-T cell therapy infusion	Coronary artery disease
	High lipids
	Aortic stenosis
	High baseline creatinine
	High troponin level

CAR-T Cell Therapy’s Cardiotoxicity

There are several heart complications associated with CAR-T therapy such as hypotension, left ventricular dysfunction, and shock. There are several studies that evaluate heart complications in children and adults [1].

Cardiotoxicities in Pediatric and Young Adult Patients

Fitzgerald et al.'s retrospective study in 2017 evaluated 39 children and young adult patients who were treated for refractory acute lymphocytic leukemia using CAR-T therapy. Reports of the study showed that more than one-third of subjects had heart complications such as shock. 14 patients of the 39 had hypotension according to the study. Moreover, 10 patients out of the 14 patients had a vasogenic shock that required multiple vasogenic medications [1,7,17,18]. 

In 2018, Burstein et al.'s retrospective study on 98 children and young adult patients who took CAR-T cell therapy for relapsed acute lymphocytic leukemia showed that 24 patients had hypotension that required inotropic medications. Of the 24 patients, 10 patients had new findings of left ventricular systolic dysfunction, which was resolved within six months in six of the 10 patients. Also, six patients of the 24 reported new ST segment changes on the ECG [1,5,7,19].

 In July 2020, Shalabi et al.'s retrospective study evaluated 52 children and young adult subjects who received CAR-T therapy. The study researched the effects of cytokine release syndrome on heart function. In the study, heart dysfunction was defined as a 10% absolute reduction in left ventricular ejection fraction from baseline or new-onset Grade two or above left ventricular dysfunction. In the study, six subjects who had Grade two or above CRS had cardiac dysfunction with a 12% decreased ejection fraction. Out of the six subjects, four subjects had cardiac function resolution 28 days post-CAR-T cell infusion and the remaining two patients had cardiac function resolution in three months [1,2,5,20].

In the ELIANA phase II clinical trial for CAR-T therapy given to refractory acute lymphocytic leukemia B cell type pediatric patients, 22 subjects reported hypotension, three subjects had left ventricular dysfunction, three subjects had tachycardia, two patients had heart failure, and three subjects had heart arrest [5,14,21]. 

Table 5 shows the heart complications associated with different studies [1,2,5,7,14,17-21].

**Table 5:** Different heart complications reported with different pediatric studies Sources:  [1,2,5,7,14,17-21] CAR: Chimeric antigen receptor; CD: Cluster of differentiation; CRS: Cytokines release syndrome

First author, Year	Study name	Study type	Outcome
Patel et al., 2021 [1]	Cardiovascular toxicities of CAR T-cell therapy	Review	Fitzgerald et al.'s retrospective study reported that 14 patients had hypotension and 10 of them had a vasogenic shock.
Lefebvre et al., 2020 [7]	Cardiovascular effects of CAR-T cell therapy: a retrospective study	Retrospective study	
Ghosh et al., 2020. [17]	CAR T cell therapy-related cardiovascular outcomes and management: systemic disease or direct cardiotoxicity?	Review	
Fitzgerald et al., 2017 [18]	Cytokine release syndrome after chimeric antigen receptor T cell therapy for acute lymphoblastic leukemia	Clinical trial	
Patel et al., 2021 [1]	Cardiovascular toxicities of CAR T-cell therapy	Review	Burstein et al.'s retrospective study reported that 24 patients had hypotension and required inotropic medications, 10 patients had new findings of left ventricular systolic dysfunction, and six patients of the 24 reported new ST segment changes on the ECG.
Burns et al., 2021 [5]	Cardiotoxicity associated with anti-CD19 chimeric antigen receptor T-cell (CAR-T) therapy: recognition, risk factors, and management	Review	
Lefebvre et al., 2020 [7]	Cardiovascular effects of CAR-T cell therapy: a retrospective study	Retrospective study	
Burstein et al., 2018 [19]	Cardiac profile of chimeric antigen receptor T cell therapy in children: a single-institution experience	Retrospective study	
Patel et al., 2021 [1]	Cardiovascular Toxicities of CAR T-cell therapy	Review	Shalabi et al.'s retrospective study reported that six patients who had grade II or above CRS had cardiac dysfunction.
Totzeck et al., 2022 [2]	Cardiotoxicity from chimeric antigen receptor-T cell therapy for advanced malignancies	Review	
Burns et al., 2021 [5]	Cardiotoxicity associated with anti-CD19 chimeric antigen receptor T-cell (CAR-T) therapy: recognition, risk factors, and management	Review	
Shalabi et al., 2020 [20]	Impact of cytokine release syndrome on cardiac function following CD19 CAR-T cell therapy in children and young adults with hematological malignancies	retrospective study	
Burns et al., 2021 [5]	Cardiotoxicity associated with anti-CD19 chimeric antigen receptor T-cell (CAR-T) therapy: recognition, risk factors, and management	Review	ELIANA phase II clinical trial reported that 22 patients had hypotension, three patients had left ventricular dysfunction, three patients had tachycardia, two patients had heart failure, and three patients had heart arrest.
Stein-Merlob et al., 2021 [14]	Cardiotoxicities of novel cancer immunotherapies	Review	
Buechner et al., 2021 [21]	Practical guidelines for monitoring and management of coagulopathy following tisagenlecleucel CAR T-cell therapy	Multi-center study	

Cardiotoxicities in Adult Patients

JULIET phase II clinical trial for tisagenlecleucel CAR-T cell therapy treating refractory diffuse large B-cell lymphoma had 93 patients followed in efficacy analysis set. 29 patients of the 93 had hypotension and eight patients with hypotension required inotropic medications [5,22,23].

ZUMA-1 phase II clinical trial for axicabtagene ciloleucel CAR-T therapy treating patients with large B-cell lymphoma had 101 patients in the efficacy analysis. Of the 101 patients, 60 had hypotension, 14 had hypotension requiring inotropic medication, and 39 patients had tachycardia [5,6,24].

ZUMA-2 phase II clinical trial for brexucabtagene autoleucel CAR-T therapy treating patients with mantle-cell lymphoma had 68 patients in the efficacy analysis. Of the 68 patients 35 had hypotension, 15 had hypotension requiring inotropic medication and 21 patients had tachycardia. None of the patients who participated in JULIET, ZUMA-1, or ZUMA-2 trials had left ventricular dysfunction, cardiac arrest, or cardiac failure, and all the CAR-T cell therapies associated with the three trials were approved by the FDA [5,6,25,26].

Also, there were multiple retrospective studies that tracked the cardiotoxicities associated with CAR-T therapy. Alvi et al.'s retrospective study was the first study in 2019. Lefebvre et al. and Gantra et al. were other retrospective studies as well. Table 6 shows the cardiotoxicities found in the retrospective studies [2,4-7,27]. Additionally, in Goldman et al.'s retrospective study, which has 2,657 patients, 74 reported tachyarrhythmia, 69 reported cardiomyopathy, and 11 pericardial effusion [26]. One of the important findings regarding adult cardiac adverse that the events do not always get resolved compared to pediatrics and young adult population [5].

**Table 6:** Reported cardiotoxicities in three retrospective studies for CAR-T therapy Sources: [2,4-7,27] CAR: Chimeric antigen receptor; CD: Cluster of differentiation

First Author, Year	Study name	Study type	Outcome
Totzeck et al., 2022 [2]	Cardiotoxicity from chimeric antigen receptor-T cell therapy for advanced malignancies.	Review	This study reported that six patients had hypotension, six patients had ventricular systolic dysfunction, six patients had tachycardia, five patients had arrhythmia, and six patients had heart arrest or death.
Burns et al., 2021 [5]	Cardiotoxicity associated with anti-CD19 chimeric antigen receptor T-cell (CAR-T) therapy: recognition, risk factors, and management	Review	
Alvi et al., 2019 [6]	Cardiovascular events among adults treated with chimeric antigen receptor T-cells (CAR-T)	Retrospective study	
Stein-Merlob et al., 2021 [27]	Immunotherapy-associated cardiotoxicity of immune checkpoint inhibitors and chimeric antigen receptor T cell therapy: diagnostic and management challenges and strategies	Review	
Totzeck et al., 2022 [2]	Cardiotoxicity from chimeric antigen receptor-T cell therapy for advanced malignancies.	Review	Lefebvre retrospective study reported that 33 patients had hypotension, 21 patients had ventricular systolic dysfunction, 13 patients had arrhythmia, two patients had heart arrest or death, and 29 patients had high troponin level which illustrate myocyte damage.
Burns et al., 2021 [5]	Cardiotoxicity associated with anti-CD19 chimeric antigen receptor T-cell (CAR-T) therapy: recognition, risk factors, and management	Review	
Lefebvre et al., 2020 [7]	Cardiovascular effects of CAR-T cell therapy: a retrospective study	Retrospective study	
Stein-Merlob et al., 2021 [27]	Immunotherapy-asociated cardiotoxicity of immune checkpoint inhibitors and chimeric antigen receptor T cell therapy: diagnostic and management challenges and strategies	Review	
Totzeck et al., 2022 [2]	Cardiotoxicity from chimeric antigen receptor-T cell therapy for advanced malignancies	Review	This study reported that five patients had hypotension, 12 patients had ventricular systolic dysfunction, and three patients had heart arrest or death.
Ganatra et al., 2019 [4]	Chimeric antigen receptor T-cell therapy for cancer and heart: JACC council perspectives	Retrospective study	
Burns et al., 2021 [5]	Cardiotoxicity associated with anti-CD19 chimeric antigen receptor T-cell (CAR-T) therapy: recognition, risk factors, and management	Review	

Monitoring Cardiotoxicities

Due to the complications that CAR-T cell therapy results in, monitoring the adverse events are necessary. As the therapy is new, the following steps were recently added to monitor patients who are getting medicated as follows. The patient is to be monitored on days zero, three, and seven from the infusion of CAR-T cell therapy. If CRS happened in Grade one, no further heart monitoring is needed. If the syndrome happens in Grade two or above, the patient's troponin gets measured daily with measuring N-terminal-pro hormone vrain natriuretic peptide (NT-proBNP) on Days zero, three, and seven, telemetry monitoring continuously, and transtheoretical echo with stain. Then, the patient needs to be followed up with in one month. Additionally, all patients with a CRS grade need a cardiac follow-up every three months [1,4,17,28].

Limitations

The inclusion in the review was limited to the population of patients receiving CAR-T cell therapy for hematologic malignancies. Also, the publications included in the review were limited to full-text articles published within the last ten years, clinical trials, meta-analyses, randomized controlled trials, reviews, and systematic reviews, excluding non-English articles. There are only three retrospective studies in pediatrics and four retrospective studies in adults to monitor the cardiotoxicity associated with CAR-T cell therapy. The sample size retrospective studies were small.

## Conclusions

CAR-T cell therapy has been an epic novel treatment for hematologic malignancies. However, there were several dangerous toxicities associated with the therapy. One of the important toxicities is cardiotoxicity, which is associated with the CRS. The cardiotoxicity happened in pediatrics, young adult, and adult populations. Even though the cardiotoxicity was dangerous, and life-threatening in some cases, there were not many systematic reviews on the topic. This systematic review shed light on the cardiotoxicity that happens with CAR-T cell therapy. In the review, data from several retrospective studies and reviews were used to illustrate the cardiac adverse events that happen in adults and children. Future retrospective and prospective studies to monitor the cardiac adverse events associated with CAR-T cell therapy are encouraged. The studies will give a clearer image of the cardiotoxicity associated with CAR-T cell therapy, its severity, and its impact on patients’ overall well-being.
